# Genetic Features of Antarctic *Acinetobacter radioresistens* Strain A154 Harboring Multiple Antibiotic-Resistance Genes

**DOI:** 10.3389/fcimb.2019.00328

**Published:** 2019-09-13

**Authors:** Andrés Opazo-Capurro, Paul G. Higgins, Julia Wille, Harald Seifert, Camila Cigarroa, Paulina González-Muñoz, Mario Quezada-Aguiluz, Mariana Domínguez-Yévenes, Helia Bello-Toledo, Luis Vergara, Gerardo González-Rocha

**Affiliations:** ^1^Laboratorio de Investigación en Agentes Antibacterianos (LIAA), Departamento de Microbiología, Facultad de Ciencias Biológicas, Universidad de Concepción, Concepción, Chile; ^2^Millennium Nucleus for Collaborative Research on Bacterial Resistance (MICROB-R), Santiago, Chile; ^3^Institute for Medical Microbiology, Immunology and Hygiene, University of Cologne, Cologne, Germany; ^4^German Center for Infection Research (DZIF), Partner Site Bonn-Cologne, Cologne, Germany; ^5^Departamento de Ciencias Biológicas y Químicas, Facultad de Medicina y Ciencia, Universidad San Sebastián, Concepción, Chile

**Keywords:** antibiotic-resistance genes, *Acinetobacter*, Antarctica, whole-genome sequencing, resistance plasmid, ecotoxicology

## Abstract

While antibiotic-resistant bacteria have been detected in extreme environments, including Antarctica, to date there are no reports of *Acinetobacter* species isolated from this region. Here, we characterized by whole-genome sequencing (WGS) the genetic content of a single antibiotic-resistant *Acinetobacter* spp. isolate (A154) collected in Antarctica. The isolate was recovered in 2013 from soil samples at Fildes Peninsula, Antarctica, and was identified by detection of the intrinsic OXA-23 gene, and confirmed by Tetra Correlation Search (TCS) and WGS. The antibiotic susceptibility profile was determined by disc diffusion, E-test, and broth microdilution methods. From WGS data, the acquired resistome and insertion sequence (IS) content were identified by *in silico* analyses. Plasmids were studied by the alkaline lysis method followed by pulsed-field gel electrophoresis and conventional PCR. The A154 isolate was identified as *A. radioresistens* by WGS analysis and displayed >99.9 of similarity by TCS in relation with the databases. Moreover, it was resistant to ampicillin, ceftriaxone, ceftazidime, cefepime, cefotaxime, streptomycin, and kanamycin. Likewise, in addition to the intrinsic *bla*_OXA−23−like_ gene, A154 harbored the plasmid-encoded antibiotic-resistance genes *bla*_PER−2_, *tet(B), aph(3*′*)-Vla, strA*, and *strB*, as well as a large diversity of ISs. This is the first report of antibiotic-resistant *A. radioresistens* in Antarctica. Our findings show the presence of several resistance genes which could be either intrinsic or acquired in the region.

## Introduction

Antarctica possesses one of the most extreme environmental conditions on earth, where low temperatures, high levels of UV radiation, and low water availability, limit the growth of living organisms, including bacteria (Convey et al., 2008). In these ecosystems, diverse survival strategies to ward off competing microorganisms are present, including the production of antibiotics by environmental microbiota (Aronson et al., 2011). Lately, it has been determined that antibiotic production and resistance have ancient origins in nature, ranging from 2 billion years to 40 million years (Dcosta et al., 2011). In this sense, several antibiotic resistance genes (ARGs) have been identified in uncontaminated soil in Antarctica, confirming that this continent possesses a natural microbial community associated with a local resistome (Van Goethem et al., 2018). Although this natural phenomenon is old, the high use of antibiotics in human practice, as well as the rise of anthropic activity around the globe, have favored to increase the selection and dissemination of antibiotic resistant bacteria and antibiotic resistance determinants (Rabbia et al., 2016).

Although Antarctica has been historically considered as a pristine unexplored continent, the anthropogenic activity has been increasing recently, which could potentially have an impact on the resistome of the natural bacterial community (Rabbia et al., 2016). It has been demonstrated that the presence of military bases, scientific programs, and tourism, promotes the dissemination of ARGs possibly due to the introduction of human-related microorganisms (Hernandez and González-Acuña, 2016). For instance, we recently found that *Escherichia coli* isolates collected from seawater and waste-water treatment plants located in the Antarctic Treaty area showed resistance to β-lactams, aminoglycosides, tetracycline, and trimethoprim-sulphonamide, which is probably associated with discharged of inefficiently treated water emanating from waste-water plants (Rabbia et al., 2016).

*Acinetobacter radioresistens* is able to survive extreme levels of oxidative stress, desiccation, and irradiation (Touchon et al., 2014; Sacher et al., 2018) and has primarily been isolated from environmental sources (i.e., cotton, water, and soil), and has also been identified as part of the human skin microbiota of healthy people (Seifert et al., 1997), as well as from fecal samples from chickens (Ngaiganam et al., 2019). Few studies on the presence of ARGs in *A. radioresistens* are published, and the species is considered largely susceptible to antibiotics.

*A. radioresistens* isolate A154 was collected from a soil sample in Antarctica in 2013. Interestingly, it displayed an unusual resistance pattern to diverse antibiotics, including β-lactams and aminoglycosides. Hence, to further understand the genetic features of the Antarctic A154 isolate containing multiple ARGs, we report its genome sequence.

## Materials and Methods

### Bacterial Source

*Acinetobacter* spp. isolate A154 was recovered from ornitogenic soil (superficial layer) in Ardley Island (−62°12′60.00″S −58°55′59.99″W) in Fildes Peninsula, Antarctica, during a scientific expedition in 2013. Specifically, the isolate was recovered in a soil sample from a lagoon shore. Fildes Peninsula is located on King George Island and holds the largest number of scientific research bases in the Antarctic Treaty Area (Rabbia et al., 2016). The isolate was grown on trypticase soy agar incubated overnight at 30°C.

### PCR Experiments

Total DNA was extracted by the boiling water method and utilized as template for preliminary species identification by the detection of the intrinsic chromosomally-encoded *bla*_OXA−23−like_ gene.

### Susceptibility Testing

Susceptibility testing were performed by disc diffusion following the CLSI recommendations, for ampicillin (AMP, 10 μg), cefotaxime (CTX, 30 μg), ceftriaxone (CRO, 30 μg), ceftazidime (CAZ, 30 μg), cefepime (FEP, 30 μg), imipenem (IPM, 10 μg), meropenem (MEM, 10 μg), gentamicin (GEN, 10 μg), amikacin (AMK, 30 μg), kanamycin (KAN, 30 μg), streptomycin (STR, 10 μg), tetracycline (TET, 30 μg), and ciprofloxacin (CIP, 5 μg) (Clinical Laboratory Standards Institute, 2017). Minimum inhibitory concentrations (MICs) were determined by E-test and broth microdilution for AMP, CTX, IPM, MEM, GEN, AMK, and KAN (Clinical Laboratory Standards Institute, 2017).

### Whole-Genome Sequencing

Total DNA for whole-genome sequencing (WGS) was extracted using the Wizard® Genomic DNA Purification kit (Promega, USA) following the manufacturer's protocol. DNA concentration and integrity were verified using a Take3 plate (BioTek Instruments). WGS was performed by the Illumina MiSeq platform (2 × 250 bp paired end reads) with libraries prepared by the NexteraXT kit (Illumina).

### *In silico* Genome Analyses

*De novo* assembly was performed by using the SPades software version 3.9 available at the CGE server (https://cge.cbs.dtu.dk/services/SPAdes/), utilizing default values (Nurk et al., 2013). Species identification was carried out by the SpeciesFinder service, version 1.3 (https://cge.cbs.dtu.dk/services/SpeciesFinder/), which is based on the *16S rRNA* sequence. Genome annotation was accomplished using the NCBI Prokaryotic Genome Annotation Pipeline (PGAP) web-service (http://www.ncbi.nlm.nih.gov/genome/annotation_prok). Moreover, the draft genome was analyzed by the Comprehensive Genome Analysis service (https://www.patricbrc.org/app/ComprehensiveGenomeAnalysis), which incorporates the RAST tool kit (RASTtk). The genetic contexts of the contigs in which plasmid-encoded ARGs were detected, were plotted using the Artemis software, version 18.0.2 (Carver et al., 2012).

In order to establish the taxonomic relatedness of A154, we used the Tetra Correlation Search (TCS), which allows to compare the draft genome of this isolate against the genomes reference database GenomesDB (http://jspecies.ribohost.com/jspeciesws/#genomesdb), which contains >30.000 whole and draft genomes with pre-calculated overall genome relatedness indices (Richter et al., 2015). In addition, we determined the acquired resistome, insertion sequences (ISs) content and toxin-antitoxin (TA) systems through ResFinder (https://cge.cbs.dtu.dk/services/ResFinder/), RASTtk and Pathosystems Resource Integration Center (PATRIC) platforms. Plasmid replicons were searched using the PlasmidFinder tool (Carattoli et al., 2014) and the PATRIC platform. The genome of the isolate A154 was deposited at DDBJ/EMBL/GenBank under the accession number PXJD00000000.1.

### Molecular Typing

Additionally, from WGS data we extracted the seven alleles according to the Pasteur MLST scheme which were submitted to the PubMLST database (https://pubmlst.org/) in order to assign a specific sequence type (ST).

### Plasmids Studies

Plasmids were isolated by the alkaline lysis method according to a standard protocol (Kado and Liu, 1981). Afterwards, DNA concentration of the plasmids extracts were measured by spectrophotometry (BioTek Instruments) and chromosomal contamination was evaluated by detecting the *16S rDNA* gene, which is chromosomally encoded. After, ARGs previously detected in total DNA were screened using the pure DNA plasmids extracts. Moreover, we performed mating and plasmid curing experiments (Trevors, 1986; Leungtongkam et al., 2018).

## Results and Discussion

The A154 isolate was identified as *A. radioresistens* according to its *16S rRNA* sequence, and displayed >99.9 of similarity by TCS with the genome of *A. radioresistens* strain SK82 (NCBI: txid596318), thus confirming that A154 belongs to this species. Additionally, it was resistant to AMP, CRO, CTX, CAZ, FEP, KAN, and STR, intermediate to GEN, and susceptible to IMP, MEM, CIP, TET, and AMK. MICs values of AMP, CAZ, CTX, FEP, GEN, and KAN were >128, >128, 128, 32, 8, and 128 μg/mL, respectively (Table 1).

**Table 1:** Antibiotic susceptibilities and genetic features of isolate A154 from Antarctica.

**MIC (ug/mL)**						**Main genetic features**	
**AMP**	**CAZ**	**CTX**	**FEP**	**GEN**	**KAN**	**ARGs**	**ST**
>128	>128	128	32	8	128	*bla*_TEM−1B_; *bla*_SCO−1_, *aac(3)-IIa, bla*_PER−2_ (pl); *aph(3′)*-*VIa* (pl); *strA* (pl); *strB* (pl); *tet(B)* (pl)	1207

*MIC, minimum inhibitory concentration; AMP, ampicillin; CAZ, ceftazidime; CTX, cefotaxime; FEP, cefepime; GEN, gentamicin; KAN, kanamycin; ARGs, antibiotic-resistance genes; ST, sequence-type; pl, plasmid-encoded*.

From *in silico* analyses, we determined that the WGS data generated resulted in 205 contigs and a *N*_50_ contig size of 131,303 bp. Moreover, A154 genome comprised 3,448,645 bp with an average GC content of 41.5%, 3,444 coding sequences (CDS), 64 transfer RNA (tRNA) genes, and 8 ribosomal RNA (rRNA) genes.

**Figure 1:**
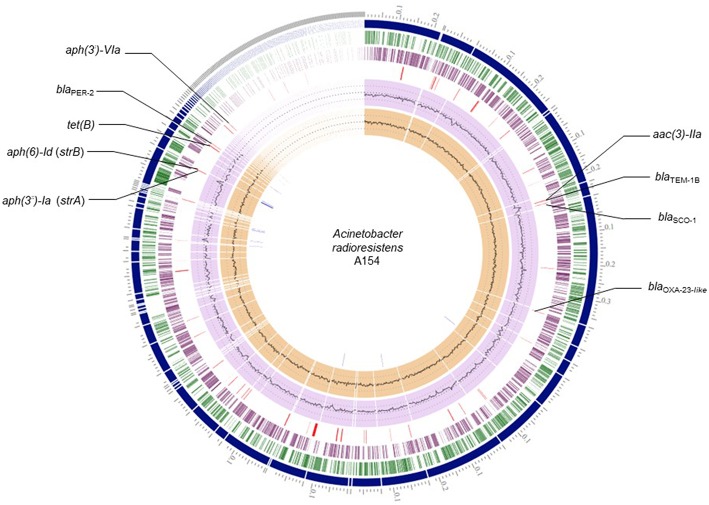
Circular representation of A154 draft genome. Circles from the outermost are as follows: contigs; forward CDS; reverse CDS; GC content; GC skew. Red lines: antibiotic-resistance genes; blue lines: transposase genes.

From RASTtk results, we determined that the resistome of A154 was comprised of aminoglycoside modifying enzymes (AMEs) encoded by *aph(3*′*)-VIa, aac(3)-IIa, strA* (*aph(3*″*)-Ia*), and *strB* (*aph(6)-Id*), as well as the β-lactamase genes *bla*_OXA−23−like_ (intrinsic to this species), *bla*_TEM−1B_, *bla*_SCO−1_ and *bla*_PER−2_, and the tetracycline-resistance gene *tet(B)* (Table 1). ARGs positions on the genome of A154 are shown in Figure 1. Interestingly, the *bla*_OXA−23−like_ allele identified in A154 did not match with any variant from the database, sharing a 98.18% identity with *bla*_OXA−565_ (accession number KY883665). SCO-1 encodes for a RTG-type carbenicillinase identified in *Escherichia coli* and *Acinetobacter* spp. (Papagiannitsis et al., 2007; Poirel et al., 2007). To the author's knowledge, the single report of this enzyme in *Acinetobacter* genus occurred in isolates collected in Argentina (Poirel et al., 2007); thus its presence could be associated to isolates from South America and nearby regions. According to Van Goethem et al. (2018), resistance determinants present in environmental soil, especially from extreme and remote ecosystems, are still underrepresented, thus our findings represent the very first description of *A. radioresistens* resistant to multiple antibiotics in this area.

Importantly, *A. radioresistens* has been rarely associated with human infections and the species is normally highly susceptible to antibiotics. In this sense, Visca et al. described a community-acquired bacteraemia caused by this species, but the isolate was susceptible to cephalosporins and aminoglycosides (Visca et al., 2001). Similarly, Poirel et al. published a study that included five *A. radioresistens* isolates that were fully susceptible to all antibiotics tested, including penicillins and carbapenems, despite the detection of *bla*_OXA−23−like_ gene (Poirel et al., 2008). On the other hand, Higgins *et al*. identified two carbapenem-resistant *A. radioresistens* isolates that were recovered from the same patient 17 days apart, which were also resistant to fluoroquinolones (Higgins et al., 2013). Additionally, two *A. radioresistens* isolates collected from companion animal infections, which harbored *bla*_IMP−1_, *sul1, aadA1*, and *aac(6)-31*, were resistant to carbapenems and fluoroquinolones (Kimura et al., 2017), but despite the presence of AMEs, the isolates were susceptible to GEN and AMK.

Our findings show that the isolate A154 was resistant to β-lactams, except carbapenems, which could be explained by the presence of the *bla*_TEM−1B_, *bla*_PER−2_, and *bla*_SCO−1_ genes. Interestingly, PER-2 corresponds to an extended-spectrum β-lactamase (Pasteran et al., 2006; Gutkind et al., 2012), while TEM-1B and SCO-1 are class A narrow-spectrum β-lactamases (Poirel et al., 2007), which could be mediating the resistance displayed against β-lactams, such as cephalosporins (Gutkind et al., 2012). These findings are particularly interesting since although β-lactamase genes are common components of the soil resistome, they are not highly abundant. Accordingly, a study of soil samples from the Mackay Glacier in Antarctica showed a very low β-lactamase abundance (Van Goethem et al., 2018). The same study also demonstrated that β-lactamase genes are more common in soil samples from regions that are more likely to have been exposed to human activity (Van Goethem et al., 2018), representing a possible source of selective pressure.

Importantly, A154 was resistant to KAN and STR, and intermediate to GEN. Although the isolate was classified as AMK susceptible, the MIC value (16 μg/mL) was only one dilution below the CLSI breakpoint for intermediate (32 μg/mL). This particular phenotype is mainly explained by the activity the APH(3′)-VIa enzyme, which was originally described in *A. baumannii* and confers resistance to AMK and KAN (Ramirez and Tolmasky, 2010). Additionally, other AME genes detected were *strA* and *strB* which confer resistance to STR (Ramirez and Tolmasky, 2010), in concordance with the observed phenotype. We also identified the *aac(3)-IIa* gene, which is related to resistance to GEN and tobramycin (TOB) (Ramirez and Tolmasky, 2010). Similarly, the identified resistome included the *tet(B)* gene, however, the isolate was susceptible to tetracycline. These inconsistencies suggest that *A. radioresistens* might need a complementary mechanism of aminoglycosides/tetracycline resistance or to regulate the expression of ARGs to develop the resistance phenotype. This assumption is reaffirmed by the presence of the intrinsic *bla*_OXA−23−like_ gene in carbapenem-susceptible *A. radioresistens*, which when present in *A. baumannii*, confers resistance to carbapenems (Evans and Amyes, 2014), when over-expressed (Higgins et al., 2013). Therefore, expression experiments of the ARGs are required in order to understand the potential influence of these determinants on the resistance level to antimicrobials in the species described.

On the other hand, A154 was assigned to the ST1207 in PubMLST (https://pubmlst.org/), which corresponds to a novel ST. This is a novel lineage which corresponds to a singleton, since is not associated to any described ST of the database. Accordingly, it is important to analyze a larger collection of isolates from regions with and without human influence in order to determine whether this ST is endemic/prevalent to this area or it was introduced. This is particularly important since we have previously determined that antibiotic-resistant *E. coli* isolates in Antarctica are more frequent in regions with a significant anthropic influence (Rabbia et al., 2016). In the case of A154, it was collected from the same region of the study mentioned above, thus the local microbiome and resistome are probably affected by human activity. However, since the soil from which the isolate was recovered corresponded to surface soil, which is determined by the influence of organic matter from animals, it is possible that it could be carried by migrating birds (Segawa et al., 2013), which might be playing an important role in the genetic contamination of this region.

**Figure 2:**
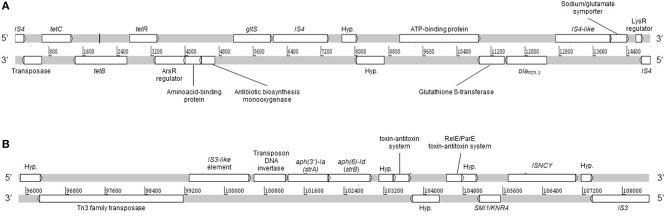
Linear map of A154 contigs 35 **(A)** and 10 **(B)**. DNA strands sense and anti-sense are represented. Hyp., hypothetical protein. The linear representation was generated by using Artemis software.

Moreover, no plasmid replicons were detected by PlasmidFinder and PATRIC. However, we detected a large plasmid of ca. 280 kb which was not detected *in silico* from WGS (data not shown). Moreover, the plasmid could not be transferred by conjugation and neither cured by temperature. The latter could be explained by the presence of the type II toxin-antitoxin systems RelB/DinJ (accession number PSD36295.1) and RelE/ParE (accession number PSD36025.1), which were detected from WGS. Interestingly, the RelE/ParE genes were located adjacent to *strA* and *strB* genes (Figure 2), which suggests that both are contained in the same plasmid. The toxin-antitoxin systems are defined as plasmid maintenance systems present in almost all free-living bacteria (Kang et al., 2018), which could confer stability against curing agents. Conjugation experiments under different conditions should be performed in order to determine the ability of the plasmid to be mobilized.

Remarkably, a large diversity of IS-family transposases was found on the genomes of A154. Specifically, its IS content was composed by fifteen copies of IS/IS1182 transposase, twelve of IS6, 11 of IS30, eight of IS4, four of ISL3, two each of IS3-like and IS5-like, and a single copy each of IS982, IS200/IS605, ISNCY, IS481, and IS1. IS-elements are capable of mobilizing and controlling the expression of ARGs, among other functions, therefore the isolate has an important potential to acquire/disseminate and over-express resistance determinants (Siguier et al., 2014). Notably, the *bla*_PER−2_; *aph(3*′*)-VIa*; *strA*; *strB*; *tet(B)* were identified in the plasmid extract obtained from alkaline lysis, and are also located in the same region of the genome in which diverse ISs were also located (Figure 1). This suggests that they could has been incorporated by A154 by horizontal-gene transfer mediated by plasmids and/or IS-elements. As shown in Figure 2, the plasmid-encoded ARGs were located in different contigs from WGS, in which also several IS-elements were located, reaffirming the potential role of these mobile-genetic elements in their mobilization. The contig in which *aph(3*′*)-VIa* is located, was not represented since it was contained in a short contig, which harbored only two genes.

An important limitation of our study is the Illumina platform generates short reads, making it difficult to determine the entire sequence and structure of the plasmid contained by A154 (Roosaare et al., 2018). In consequence, the use of this sequencing technology to predict plasmids (i.e., by PlasmidFinder) is limited, being only possible to search for the incompatibility groups detected in the genome which are deposited on the databases. A potential solution for this problem, is to isolate the plasmid and sequence it or use new sequencing technologies, such as Oxford Nanopore Technology (ONT) (Lemon et al., 2017).

In conclusion, our results represent the first description of *A. radioresistens* in Antarctica containing several ARGs, in an isolate recovered from a region under both human and animal influences, thus these factors could be contributing to the introduction of ARGs in Antarctica. These findings contribute to better understand the knowledge of the biology of *Acinetobacter* as well as to add more data about the content of ARGs in the region.

## Data Availability

The raw data supporting the conclusions of this manuscript will be made available by the authors, without undue reservation, to any qualified researcher.

## Author Contributions

AO-C, PG-M, PH, and JW contributed to the acquisition, analysis, and interpretation of the data. AO-C, CC, PG-M, and MQ-A made important contributions to the design of the work. HS, MD-Y, HB-T, LV, and GG-R provided approval for publication of the content and contributed drafting the work and critically revisiting the manuscript.

### Conflict of Interest Statement

The authors declare that the research was conducted in the absence of any commercial or financial relationships that could be construed as a potential conflict of interest.

## References

[B1] Aronson R. B. Thatje S. Mcclintock J. B. Hughes K. A. (2011). Anthropogenic impacts on marine ecosystems in Antarctica. Ann. N. Y. Acad. Sci. 1223, 82–107. 10.1111/j.1749-6632.2010.05926.x 21449967

[B2] Carattoli A. Zankari E. Garciá-Fernández A. Larsen M. V. Lund O. Villa L.. (2014). *In silico* detection and typing of plasmids using plasmidfinder and plasmid multilocus sequence typing. Antimicrob. Agents Chemother. 58, 3895–3903. 10.1128/AAC.02412-14 24777092 PMC4068535

[B3] Carver T. Harris S. R. Berriman M. Parkhill J. McQuillan J. A. (2012). Artemis: An integrated platform for visualization and analysis of high-throughput sequence-based experimental data. Bioinformatics 28, 464–469. 10.1093/bioinformatics/btr703 22199388 PMC3278759

[B4] Clinical and Laboratory Standards Institute (2017). CLSI. Performance Standards for Antimicrobial Susceptibility Testing. 27th Edn. CLSI supplement M100. Wayne, PA: Clinical and Laboratory Standards Institute.

[B5] Convey P. Gibson J. A. E. Hillenbrand C. D. Hodgson D. A. Pugh P. J. A. Smellie J. L.. (2008). Antarctic terrestrial life–challenging the history of the frozen continent? Biol. Rev. 83, 103–117. 10.1111/j.1469-185X.2008.00034.x 18429764

[B6] Dcosta V. M. King C. E. Kalan L. Morar M. Sung W. W. L. Schwarz C. (2011). Antibiotic resistance is ancient. Nature 477, 457–461. 10.1038/nature10388 21881561

[B7] Evans B. A. Amyes S. G. B. (2014). OXA β-lactamases. Clin. Microbiol. Rev. 27, 241–263. 10.1128/CMR.00117-13 24696435 PMC3993105

[B8] Gutkind O. G. Di Conza J. Power P. Radice M. (2012). β-lactamase-mediated resistance: a biochemical, epidemiological and genetic overview. Curr. Pharm. Des. 19, 164–208. 10.2174/13816128130202 22894615

[B9] Hernandez J. González-Acuña D. (2016). Anthropogenic antibiotic resistance genes mobilization to the polar regions. Infect. Ecol. Epidemiol. 12:32112 10.3402/iee.v6.32112 PMC5149653 27938628

[B10] Higgins P. G. Zander E. Seifert H. (2013). Identification of a novel insertion sequence element associated with carbapenem resistance and the development of fluoroquinolone resistance in *Acinetobacter radioresistens*. J. Antimicrob. Chemother. 68, 720–722. 10.1093/jac/dks446 23139290

[B11] Kado C. Liu S. (1981). Rapid procedure for detection and isolation of large and small plasmids. J. Bacteriol. 145, 1365–1373. 7009583 10.1128/jb.145.3.1365-1373.1981 PMC217141

[B12] Kang S. M. Kim D. H. Jin C. Lee B. J. (2018). A systematic overview of type II and III toxin-antitoxin systems with a focus on druggability. Toxins (Basel) 10:E515. 10.3390/toxins10120515 30518070 PMC6315513

[B13] Kimura Y. Miyamoto T. Aoki K. Ishii Y. Harada K. Watarai M.. (2017). Analysis of IMP-1 type metallo-β-lactamase-producing *Acinetobacter radioresistens* isolated from companion animals. J. Infect. Chemother. 23, 655–657. 10.1016/j.jiac.2017.03.011 28408304

[B14] Lemon J. K. Khil P. P. Frank K. M. Dekker J. P. (2017). Rapid nanopore sequencing of plasmids and resistance gene detection in clinical isolates. J. Clin. Microbiol. 55, 3530–3543. 10.1128/JCM.01069-17 29021151 PMC5703817

[B15] Leungtongkam U. Thummeepak R. Tasanapak K. Sitthisak S. (2018). Acquisition and transfer of antibiotic resistance genes in association with conjugative plasmid or class 1 integrons of *Acinetobacter baumannii*. PLoS ONE 13:e0208468 10.1371/journal.pone.0208468 30521623 PMC6283642

[B16] Ngaiganam E. P. Rolain J.-M. Diene S. M. (2019). Detection of a new variant of OXA-23 carbapenemase in *Acinetobacter radioresistens* isolates from urban animals in Marseille, France. J. Glob. Antimicrob. Resist. 16, 178–180. 10.1016/j.jgar.2019.01.021 30685462

[B17] Nurk S. Bankevich A. Antipov D. Gurevich A. A. Korobeynikov A. Lapidus A.. (2013). Assembling single-cell genomes and mini-metagenomes from chimeric MDA products. J. Comput. Biol. 20, 714–737. 10.1089/cmb.2013.0084 24093227 PMC3791033

[B18] Papagiannitsis C. C. Loli A. Tzouvelekis L. S. Tzelepi E. Arlet G. Miriagou V. (2007). SCO-1, a novel plasmid-mediated class A β-lactamase with carbenicillinase characteristics from *Escherichia coli*. Antimicrob. Agents Chemother. 51, 2185–2188. 10.1128/AAC.01439-06 17353248 PMC1891400

[B19] Pasteran F. Rapoport M. Petroni A. Faccone D. Corso A. Galas M.. (2006). Emergence of PER-2 and VEB-1a in *Acinetobacter baumannii* strains in the Americas. Antimicrob. Agents Chemother. 50, 3222–3224. 10.1128/AAC.00284-06 16940137 PMC1563550

[B20] Poirel L. Corvec S. Rapoport M. Mugnier P. Petroni A. Pasteran F.. (2007). Identification of the novel narrow-spectrum β-lactamase SCO-1 in *Acinetobacter* spp. from Argentina. Antimicrob. Agents Chemother. 51, 2179–2184. 10.1128/AAC.01600-06 17420213 PMC1891420

[B21] Poirel L. Figueiredo S. Cattoir V. Carattoli A. Nordmann P. (2008). *Acinetobacter radioresistens* as a silent source of carbapenem resistance for *Acinetobacter* spp. Antimicrob. Agents Chemother. 52, 1252–1256. 10.1128/AAC.01304-07 18195058 PMC2292503

[B22] Rabbia V. Bello-Toledo H. Jiménez S. Quezada M. Domínguez M. Vergara L. (2016). Antibiotic resistance in *Escherichia coli* strains isolated from Antarctic bird feces, water from inside a wastewater treatment plant, and seawater samples collected in the Antarctic Treaty area. Polar Sci. 10, 123–131. 10.1016/j.polar.2016.04.002

[B23] Ramirez M. S. Tolmasky M. E. (2010). Aminoglucoside modifing enzymes. Drug Resist. Updat. 13, 151–171. 10.1016/j.drup.2010.08.003 20833577 PMC2992599

[B24] Richter M. Rosselló-Móra R. Oliver Glöckner F. Peplies J. (2015). JSpeciesWS: A web server for prokaryotic species circumscription based on pairwise genome comparison. Bioinformatics 32, 929–931. 10.1093/bioinformatics/btv681 26576653 PMC5939971

[B25] Roosaare M. Puustusmaa M. Möls M. Vaher M. Remm M. (2018). PlasmidSeeker: identification of known plasmids from bacterial whole genome sequencing reads. PeerJ 6:e4588. 10.7717/peerj.4588 29629246 PMC5885972

[B26] Sacher J. C. Yee E. Szymanski C. M. Miller W. G. (2018). Complete genome sequence of *Acinetobacter radioresistens* strain LH6, a multidrug-resistant bacteriophage-propagating strain. Microbiol. Resour. Announc. 7:e00929–18. 10.1128/MRA.01317-18 30533885 PMC6256452

[B27] Segawa T. Takeuchi N. Rivera A. Yamada A. Yoshimura Y. Barcaza G.. (2013). Distribution of antibiotic resistance genes in glacier environments. Environ. Microbiol. Rep. 5, 127–134. 10.1111/1758-2229.12011 23757141

[B28] Seifert H. Dijkshoorn L. Gerner-Smidt P. Pelzer N. Tjernberg I. Vaneechoutte M. (1997). Distribution of *Acinetobacter* species on human skin: Comparison of phenotypic and genotypic identification methods. J. Clin. Microbiol. 35, 2819–2825. 10.1128/CMR.00064-16 9350741 PMC230069

[B29] Siguier P. Gourbeyre E. Chandler M. (2014). Bacterial insertion sequences: their genomic impact and diversity. FEMS Microbiol. Rev. 38, 865–891. 10.1111/1574-6976.12067 24499397 PMC7190074

[B30] Touchon M. Cury J. Yoon E. J. Krizova L. Cerqueira G. C. Murphy C.. (2014). The genomic diversification of the whole *Acinetobacter* genus: origins, mechanisms, and consequences. Genome Biol. Evol. 6, 2866–2882. 10.1093/gbe/evu225 25313016 PMC4224351

[B31] Trevors J. T. (1986). Plasmid curing. FEMS Microbiol. Rev. 32, 149–157.

[B32] Van Goethem M. W. Van Pierneef R. Bezuidt O. K. I. Peer Y. Van De Cowan D. A. Makhalanyane T. P. (2018). A reservoir of ‘historical' antibiotic resistance genes in remote pristine Antarctic soils. Microbiome 6:40. 10.1186/s40168-018-0424-5 29471872 PMC5824556

[B33] Visca P. Petrucca A. De Mori P. Festa A. Boumis E. Antinori A.. (2001). Community-acquired *Acinetobacter radioresistens* bacteremia in an HIV-positive patient. Emerg. Infect. Dis. 7, 1032–1035. 10.3201/eid0706.010621 11747736 PMC2631918

